# VEGF Inhibitors Improve Survival Outcomes in Patients with Liver Metastases across Cancer Types—A Meta-Analysis

**DOI:** 10.3390/cancers15205012

**Published:** 2023-10-16

**Authors:** Jordan W. Conway, Jorja Braden, Serigne N. Lo, Richard A. Scolyer, Matteo S. Carlino, Alexander M. Menzies, Georgina V. Long, Ines Pires da Silva

**Affiliations:** 1 Melanoma Institute Australia, The University of Sydney, 40 Rocklands Rd, North Sydney, NSW 2065, Australia; 2 Faculty of Medicine and Health, The University of Sydney, Sydney, NSW 2050, Australia; 3 Charles Perkins Centre, The University of Sydney, Sydney, NSW 2050, Australia; 4 Royal Prince Alfred Hospital, Sydney, NSW 2050, Australia; 5 NSW Health Pathology, Sydney, NSW 2099, Australia; 6 Crown Princess Mary Cancer Centre, Westmead and Blacktown Hospitals, Sydney, NSW 2148, Australia; 7 Royal North Shore Hospital, Sydney, NSW 2065, Australia; 8 Mater Hospital, Sydney, NSW 2060, Australia

**Keywords:** drug resistance, immunotherapy resistance, liver metastases, meta-analysis, overall survival, progression-free survival, randomized controlled trials, VEGF inhibitor

## Abstract

**Simple Summary:**

The liver is a common site of metastasis across multiple solid organ malignancies. Liver metastases are a known site of treatment resistance, regardless of the site of primary tumour, and their presence is associated with a poor prognosis. This meta-analysis of 4445 patients from 25 randomized controlled trials demonstrated that the addition of vascular endothelial growth factor inhibitors to standard of care improved survival in patients with liver metastases across cancer types. This study highlights the efficacy of vascular endothelial growth factor inhibitors in liver metastases and suggests a treatment approach for clinicians with a focus on sites of metastasis rather than the established primary-specific approach.

**Abstract:**

Background: Liver metastases are associated with poor prognosis across cancers. Novel treatment strategies to treat patients with liver metastases are needed. This meta-analysis aimed to assess the efficacy of vascular endothelial growth factor inhibitors in patients with liver metastases across cancers. Methods: A systematic search of PubMed, Cochrane CENTRAL, and Embase was performed between January 2000 and April 2023. Randomized controlled trials of patients with liver metastases comparing standard of care (systemic therapy or best supportive care) with or without vascular endothelial growth factor inhibitors were included in the study. Outcomes reported included progression-free survival and overall survival. Results: A total of 4445 patients with liver metastases from 25 randomized controlled trials were included in this analysis. The addition of vascular endothelial growth factor inhibitors to standard systemic therapy or best supportive care was associated with superior progression-free survival (HR = 0.49; 95% CI, 0.40–0.61) and overall survival (HR = 0.83; 95% CI, 0.74–0.93) in patients with liver metastases. In a subgroup analysis of patients with versus patients without liver metastases, the benefit with vascular endothelial growth factor inhibitors was more pronounced in the group with liver metastases (HR = 0.44) versus without (HR = 0.57) for progression-free survival, but not for overall survival. Conclusion: The addition of vascular endothelial growth factor inhibitors to standard management improved survival outcomes in patients with liver metastasis across cancers.

## 1. Introduction

The liver is a common site of metastasis, and the presence of liver metastases is a poor prognostic factor in several cancers [1,2]. Furthermore, in melanoma, non-small cell lung cancer (NSCLC), and renal cell carcinoma (RCC), the presence of liver metastases has been associated with poorer response and survival in patients treated with immunotherapy [1,3,4,5].

Hepatocellular carcinoma (HCC) is the most common primary liver cancer and is known to be resistant to chemotherapy [6,7]. Nevertheless, in the last decade, targeting angiogenesis with vascular endothelial growth factor (VEGF) inhibitors (VEGFi) has improved clinical outcomes in patients with advanced HCC [8,9,10]. Additionally, immunotherapy as monotherapy for HCC has seen modest responses [11]; however, combination immunotherapy with VEGFi in recent years has demonstrated more robust responses [10]. HCC is characterized by an immunosuppressive, hypoxic, and highly vascularized tumour microenvironment [12,13]. In the presence of oxygen, hypoxia-inducible factor-1α (HIF1α) is degraded; however, in a hypoxic microenvironment (e.g., in the context of an aggressive tumour), HIF1α binds to hypoxia-inducible factor-1β (HIF1β), leading to the transcription of target genes, including VEGF, which plays a key role in angiogenesis [14]. A high level of VEGF in the plasma is a poor prognostic feature in several cancer types, and the blockade of the VEGF–VEGFR signalling pathway has demonstrated the significant improvement of clinical outcomes in some cancers [15] besides HCC [8,9,10], including renal cell carcinoma (RCC) [16,17,18] and colorectal cancer (CRC) [19,20]. Over the years, multiple drugs have been developed to block the VEGF–VEGFR signalling pathway. These encompass different classes of drugs, including monoclonal antibodies against VEGF (e.g., bevacizumab) [21], and tyrosine kinase inhibitors (TKIs), which target multiple pathways, including VEGF–VEGFR (e.g., sunitinib) [22,23], amongst others.

The liver is the most common site of metastasis in CRC, with 25–50% of patients presenting with liver metastases at the time of diagnosis [24], and the addition of bevacizumab to FOLFOX/CAPOX (5-fluorouracil (5-FU) or capecitabine in combination with oxaliplatin) or FOLFIRI/CAPIRI (5-FU or capecitabine in combination with irinotecan) has shown significant improvement in objective response rate (ORR) and survival in these patients [19]. Whether this strategy is also effective in liver metastases in patients with other cancer types is unknown.

In this study, we aimed to assess the efficacy of VEGFi in cancer patients with liver metastases in a meta-analysis including randomized–controlled clinical trials (RCTs) testing the efficacy of VEGFi, regardless of primary cancer site. We also compared VEGFi efficacy in patients with versus without liver metastases.

## 2. Methods

### 2.1. Search Strategy and Selection Criteria

Systematic searches of PubMed, Cochrane CENTRAL, and Embase were conducted from 1 January 2000 to 30 April 2023, based on the following criteria: Population, stage IV solid organ malignancy with liver metastasis. Hepatocellular carcinoma was excluded. Intervention, backbone of systemic therapy (chemotherapy and/or immunotherapy and/or non-VEGFi targeted therapy) or best supportive care (BSC) with a VEGFi (tyrosine kinase inhibitors (TKI) (sunitinib, pazopanib, sorafenib, lenvatinib, vandetanib, regorafenib, cabozantinib, axitinib, cediranib, ponatinib, aflibercept, vatalanib, tivozanib, motesanib, linifanib, anlotinib, fruquintinib, nintedanib, apatinib) or monoclonal antibody (bevacizumab, ramucirumab, vanucizumab)) (Supplementary Table S1). TKIs that targeted multiple pathways were included as long as the inhibition of the VEGF–VEGFR pathway was part of the mechanism of action. Comparator, backbone of systemic therapy (chemotherapy and/or immunotherapy and/or non-VEGFi targeted therapy) or best supportive care without VEGFi. Outcome, progression-free survival (PFS) and/or overall survival (OS). Study design, published randomized clinical trial (RCTs) (Supplementary Table S2).

**Figure 1:**
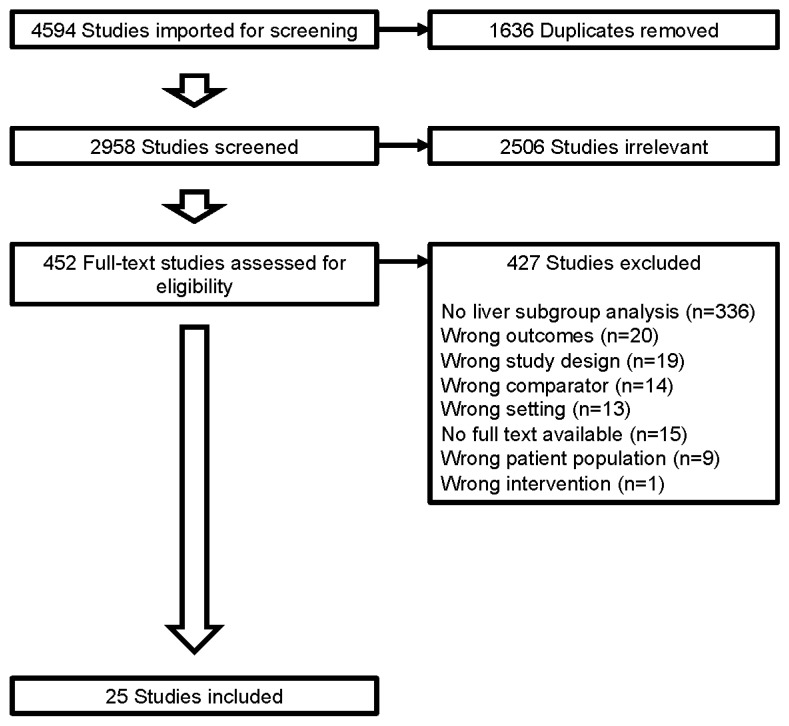
Study selection. Studies selected based on Preferred Reporting Items for Systematic Reviews and Meta-analyses (PRISMA) guidelines.

This meta-analysis followed the Preferred Reporting Items for Systematic Reviews and Meta-analysis (PRISMA) guidelines (Figure 1) and was registered with the International Platform of Registered Systematic Review and Meta-analysis Protocols (registration number; INPLASY 202390034).

### 2.2. RCT Quality Assessment and Mitigation of Bias

Study inclusion criteria were established prior to commencing database searches. Three authors (JWC, JB, and IPdS) conducted independent database searches. Each study underwent individual assessment for eligibility by the respective reviewing authors before cross-referencing any shared final selections. Any studies not unanimously identified then underwent independent evaluation by the remaining authors to determine their eligibility for inclusion. All studies included in the final analysis were deemed to meet eligibility criteria by consensus of all authors.

We utilized the JADAD scale [25] to assess the quality of all RCTs. A score of 3 or higher defined a good-quality RCT and this cut off was required for studies to be included in this analysis (Supplementary Table S3).

### 2.3. Outcomes

The two primary outcomes of this study were the PFS and OS of the addition of VEGFi to a backbone of systemic therapy (chemotherapy and/or immunotherapy and/or non-VEGFi targeted therapy) or best supportive care, measured in terms of the PFS and/or OS differences compared with no VEGFi. Preplanned subgroups of analysis included: (a) cancer type, “colorectal cancer” and “non-colorectal cancers”; (b) backbone systemic therapy, “chemotherapy” and “non-chemotherapy”; (c) VEGFi type, “bevacizumab” and “non-bevacizumab”; (d) line of treatment, “first line” and “subsequent line”; and (e) liver metastases, “presence” and “absence”. Information extracted included the first author’s name, study name, journal and year of publication, study design, National Clinical Trials (NCT) identification number, study phase, cancer type, number of patients, lines of treatment, study drugs, and hazard ratios (HRs) with 95% CIs for OS and for PFS. In two trials, the HR for PFS was estimated from the figures on the manuscript, and in another trial, the 95% CI for HR for PFS was not provided in the manuscript.

### 2.4. Statistical Analysis

The selected studies were summarized, including the total number of patients (patients with liver metastases) and the estimated effect (HR for PFS, OS or both). The overall effects of the addition of VEGFi to standard therapy or BSC in patients with liver metastases across different cancer types were estimated by pooling HRs from individual studies using a random effect model with inverse variance. Forest plots of pooled results were generated, along with their 95% confidence intervals (CI). Heterogeneity between studies was assessed using I^2^, a statistical metric that estimates the percentage of total variation across studies [26]. An I^2^ > 75% indicates high heterogeneity between studies. The presence of potential publication bias was assessed graphically using a funnel plot.

Subgroup analyses were performed considering four pre-specified factors: cancer type, backbone systemic therapy, VEGFi type, and line of treatment. The categories within each factor are defined in the Outcomes subsection. We performed a separate analysis which included studies with data on liver metastasis versus no liver metastasis.

All statistical analyses were performed in R version 4.0.2 (R Foundation for Statistical Computing).

## 3. Results

### 3.1. Systematic Review and Characteristics

Of a total of 4594 studies identified from the literature review, 1636 duplicates were removed. A total of 2958 studies were screened (title and abstract reviewed) and 2506 were considered irrelevant due to the topic, non-randomized controlled trials, or no usable data available. A total of 452 full-text studies were assessed for eligibility, and of these, 427 studies were excluded due to no liver subgroup analysis (n = 336), wrong outcomes (n = 20), wrong study design (n = 19), wrong comparator (n = 14), wrong setting (n = 13), no full text available (n = 15), wrong patient population (n = 9), or wrong intervention (n = 1) (Figure 1).

Twenty-five RCTs were eligible and included in this meta-analysis, involving 4445 patients with liver metastases (Table 1). The 25 RCTs selected included 10 trials performed in patients with CRC [27,28,29,30,31,32,33,34,35,36], 6 trials in patients with NSCLC [37,38,39,40,41,42], 5 trials in patients with RCC or urothelial cancer [18,43,44,45,46], 1 trial in patients with pancreatic cancer [47], 1 trial in patients with gastrointestinal stromal tumour [48], 1 trial in patients with gastric cancer [49] and 1 trial in patients with melanoma [50]. The backbone of systemic therapy in these trials included chemotherapy in 13 trials, targeted therapy in 3 trials, immunotherapy in 2 trials, chemotherapy combined with targeted therapy in 1 trial, and BSC in 6 trials.

### 3.2. Progression-Free Survival Comparison

**Figure 2:**
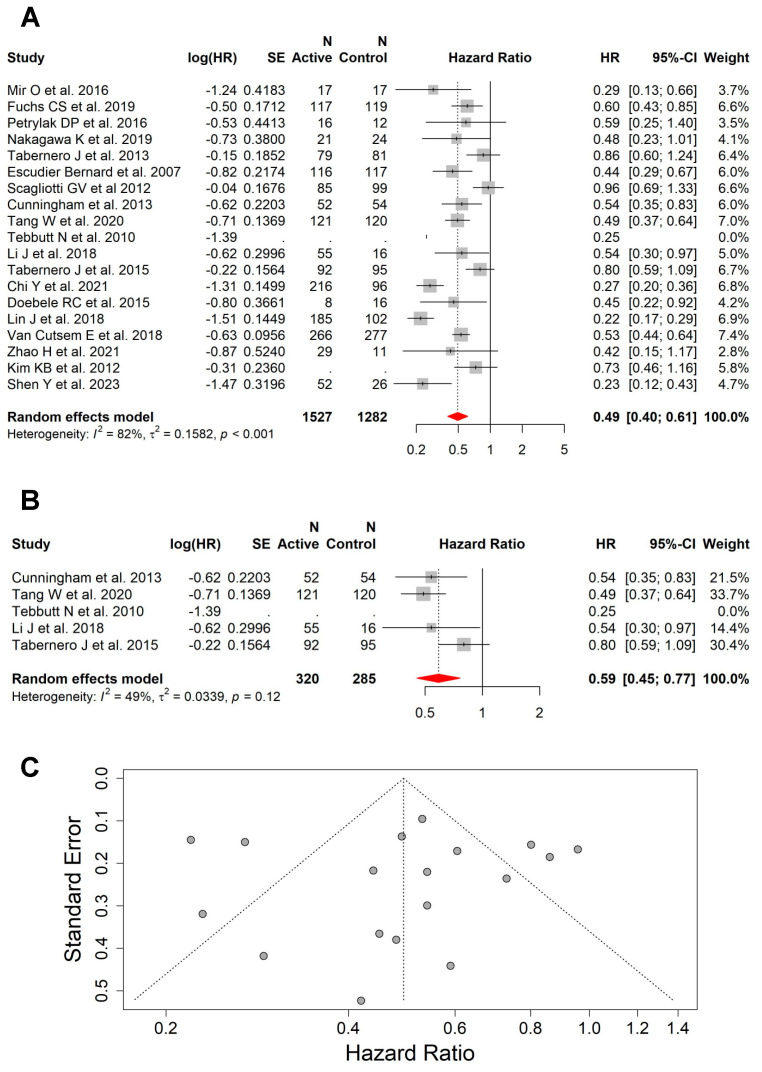
The addition of VEGFi to a backbone of systemic therapy or BSC was associated with superior PFS. (**A**) Forest plot and pooled HRs for PFS comparing the backbone systemic therapy or BSC with versus without VEGFi (HR = 0.49; 95% CI, 0.40–0.61). (**B**) Forest plot and pooled HRs for PFS comparing the backbone systemic therapy or BSC with versus without VEGFi (HR = 0.59; 95% CI, 0.45–0.77) from studies that included patients with liver as the only site of metastasis. (**C**) Funnel plot showing high heterogeneity between all studies for PFS (I^2^ = 82%, *p* < 0.001) [18,27,28,30,31,32,33,34,35,36,37,39,40,41,42,45,48,49,50].

The pooled results from 19 studies with an available HR for PFS showed that the addition of VEGFi to backbone systemic therapy or BSC was associated with superior PFS (HR = 0.49; 95% CI, 0.40–0.61) compared with no VEGFi in this group of patients (Figure 2A). The same effect was seen in the subgroup of studies (n = 5) that included patients with liver metastases only (without other sites of metastases) (HR = 0.59; 95% CI, 0.45–0.77) (Figure 2B). There was high heterogeneity between all studies for PFS (I^2^ = 82%, *p* < 0.001) (Figure 2C), but moderate heterogeneity between the studies including patients with liver metastases only (I^2^ = 49%, *p* = 0.12).

The benefit of the addition of VEGFi in PFS was seen across all preplanned subgroups, including (a) cancer type, “colorectal cancer” (HR = 0.48; 95% CI, 0.34–0.68; high heterogeneity: I^2^ = 89%, *p* < 0.0001) (Supplementary Figure S1A) and “non-colorectal cancers” (HR = 0.51; 95% CI, 0.39–0.68; high heterogeneity: I^2^ = 62%, *p* = 0.005) (Supplementary Figure S1B); (b) backbone systemic therapy, “chemotherapy” (HR = 0.63; 95% CI, 0.53–0.75; low heterogeneity: I^2^ = 25%, *p* = 0.22) (Supplementary Figure S2A) and “non-chemotherapy” (HR = 0.39; 95% CI, 0.27–0.55; high heterogeneity: I^2^ = 87%, *p* < 0.001) (Supplementary Figure S2B); (c) VEGFi type, “bevacizumab” (HR = 0.54; 95% CI, 0.44–0.67; low heterogeneity: I^2^ = 6%, *p* = 0.34) (Supplementary Figure S3A) and “non-bevacizumab” (HR = 0.48; 95% CI, 0.37–0.62; high heterogeneity: I^2^ = 85%, *p* < 0.001) (Supplementary Figure S3B); and (d) line of treatment, “first line” (HR = 0.62; 95% CI, 0.52–0.74; low heterogeneity: I^2^ = 30%, *p* = 0.18) (Supplementary Figure S4A) and “subsequent line” (HR = 0.40; 95% CI, 0.29–0.57; high heterogeneity: I^2^ = 88%, *p* < 0.001) (Supplementary Figure S4B).

### 3.3. Overall Survival Comparison

**Figure 3:**
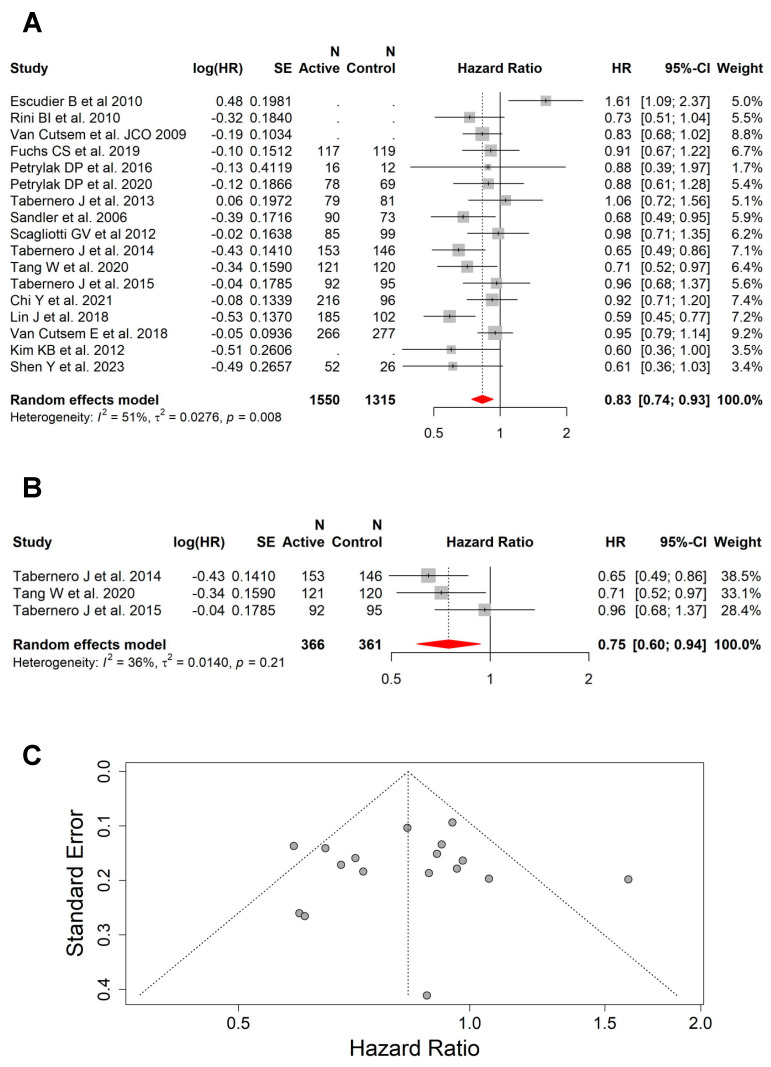
The addition of VEGFi to a backbone of systemic therapy or BSC was associated with superior OS. (**A**) Forest plot and pooled HRs for OS comparing the backbone systemic therapy or BSC with versus without VEGFi (HR = 0.83; 95% CI, 0.74–0.93). (**B**) Forest plot and pooled HRs for OS comparing the backbone systemic therapy or BSC with versus without VEGFi (HR = 0.75; 95% CI, 0.60–0.94) from studies that included patients with liver as the only site of metastasis. (**C**) Funnel plot showing moderate heterogeneity between all studies for OS (I^2^ = 51%, *p* = 0.008) [27,29,30,33,34,35,36,38,39,42,43,44,45,46,47,49,50].

This pooled analysis included 17 studies with available HR for OS, which showed that the addition of VEGFi to the backbone systemic therapy or BSC is associated with improved OS (HR = 0.83; 95% CI, 0.74–0.93) in the subset of patients with liver metastases (Figure 3A). This effect was also seen within the group of studies (n = 3) that included patients with liver metastases as the only site of disease (HR = 0.75; 95% CI, 0.60–0.94) (Figure 3B). There was moderate heterogeneity between all studies for OS (I^2^ = 51%, *p* = 0.008) (Figure 3C), but low heterogeneity between the studies including patients with liver metastases only (I^2^ = 36%, *p* = 0.21).

There was a trend (most were statistically significant) towards better OS with the addition of VEGFi in all preplanned subgroups, including: (a) cancer type, “colorectal cancer” (HR = 0.81; 95% CI, 0.69–0.96; moderate heterogeneity: I^2^ = 60%, *p* = 0.02) (Supplementary Figure S5A) and “non-colorectal cancers” (HR = 0.85; 95% CI, 0.72–1.00; moderate heterogeneity: I^2^ = 48%, *p* = 0.05) (Supplementary Figure S5B); (b) backbone systemic therapy, “chemotherapy” (HR = 0.79; 95% CI, 0.70–0.91; low heterogeneity: I^2^ = 11%, *p* = 0.34) (Supplementary Figure S6A) and “non-chemotherapy” (HR = 0.86; 95% CI, 0.71–1.05; high heterogeneity: I^2^ = 68%, *p* = 0.002) (Supplementary Figure S6B); (c) VEGFi type, “bevacizumab” (HR = 0.81; 95% CI, 0.63–1.05; high heterogeneity: I^2^ = 68%, *p* = 0.009) (Supplementary Figure S7A) and “non-bevacizumab” (HR = 0.84; 95% CI, 0.74–0.96; moderate heterogeneity: I^2^ = 41%, *p* = 0.08) (Supplementary Figure S7B); (d) line of treatment, “first line” (HR = 0.86; 95% CI, 0.73–1.02; moderate heterogeneity: I^2^ = 55%, *p* = 0.02) (Supplementary Figure S8A) and “subsequent line” (HR = 0.80; 95% CI, 0.68–0.94; moderate heterogeneity: I^2^ = 51%, *p* = 0.05) (Supplementary Figure S8B).

### 3.4. Role of Anti-VEGF in Patients with vs. without Liver Metastases

**Figure 4:**
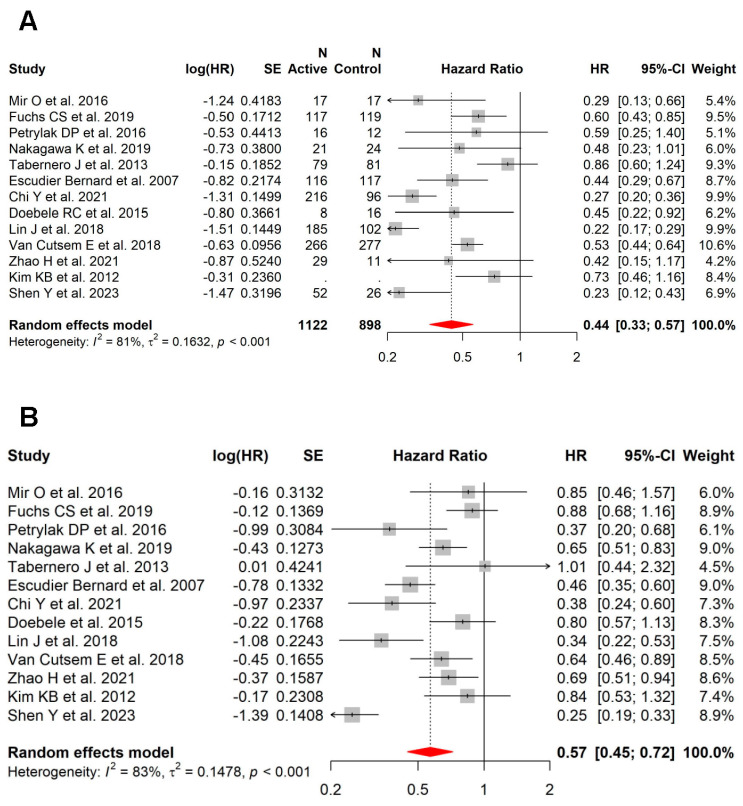
In the subset of RCTs with data on patients with and without liver metastases, the benefit with VEGFi was more pronounced in patients with liver metastases vs. those without liver metastases for PFS. (**A**) Forest plot and pooled HRs for PFS in patients with liver metastases (HR = 0.44; 95% CI, 0.33–0.57; high heterogeneity: I^2^ = 81%, *p* < 0.001). (**B**) Forest plot and pooled HRs for PFS in patients without liver metastases (HR = 0.57; 95% CI, 0.45–0.72; high heterogeneity: I^2^ = 83%, *p* < 0.001) [18,27,34,35,36,37,40,41,42,45,48,49,50].

**Figure 5:**
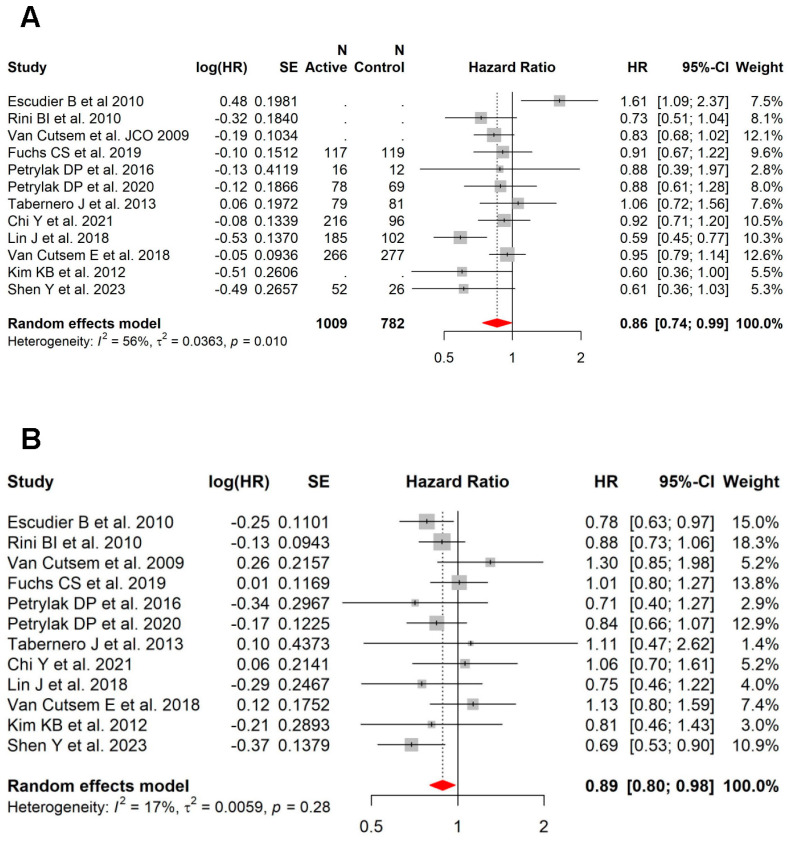
In the subset of RCTs with data on patients with and without liver metastases, the similar benefit with VEGFi was seen in patients with liver metastases vs. those without liver metastases for OS. (**A**) Forest plot and pooled HRs for OS in patients with liver metastases (HR = 0.86; 95% CI, 0.74–0.99; moderate heterogeneity: I^2^ = 56%, *p* = 0.010). (**B**) Forest plot and pooled HRs for OS in patients without liver metastases (HR = 0.89; 95% CI, 0.80–0.98; low heterogeneity: I^2^ = 17%, *p* = 0.28) [27,34,35,36,42,43,44,45,46,47,49,50].

In this meta-analysis, 17 studies also had available PFS (n = 13) and/or OS (n = 12) data on patients without liver metastases. Within this subset of studies, the benefit of the addition of VEGFi was more pronounced in patients with liver metastases (HR = 0.44; 95% CI, 0.33–0.57; high heterogeneity: I^2^ = 81%, *p* < 0.001) (Figure 4A) compared to those without liver metastases (HR = 0.57; 95% CI, 0.45–0.72; high heterogeneity: I^2^ = 83%, *p* < 0.001) for PFS (Figure 4B). In contrast, this was not seen for OS (patients with liver metastases (HR = 0.86; 95% CI, 0.74–0.99; moderate heterogeneity: I^2^ = 56%, *p* = 0.010; Figure 5A) versus patients without liver metastases (HR = 0.89; 95% CI, 0.80–0.98; low heterogeneity: I^2^ = 17%, *p* = 0.28; Figure 5B)).

## 4. Discussion

In this meta-analysis, including RCTs where patients were treated with a backbone of systemic therapy or BSC and randomized into groups with or without VEGFi, we have shown that the addition of VEGFi improved PFS and OS in patients with liver metastases across multiple cancer types. Remarkably, that benefit was more pronounced in patients with liver metastases compared to those without liver metastases, suggesting that VEGFi might be a treatment option for patients with liver metastases resistant to standard treatment.

Several studies have shown that the presence of liver metastases confers a poor prognosis across different cancers [1,2,51,52], and that patients with liver metastases are more likely to be resistant to immune checkpoint inhibitor immunotherapy compared with other sites of metastases [1,3,4,5,53,54]. This was shown in patients with metastatic melanoma, NSCLC, RCC, and urothelial cancer, treated with anti-programmed cell death 1 (anti-PD-1) monotherapy or anti-PD-1 in combination with anti-cytotoxic T-lymphocyte-associated protein 4 (anti-CTLA-4), where the presence of liver metastases was associated with a lower response and a shorter progression-free and overall survival [4,5,13,53,55]. This might be a consequence of more aggressive cancer biology that has a higher likelihood to spread to the liver, but there is also recent evidence suggesting that the presence of liver metastases negatively influences the systemic anti-tumour immune response [56,57]. The liver tolerogenic microenvironment was shown for the first time when MHC-mismatched liver allografts were grafted successfully [58]. Several immunosuppressive mechanisms have been postulated. These include the tolerogenic way of antigen presentation in the liver by Kupffer cells, stellate cells, and liver sinusoidal endothelial cells (LSECs) [59], the induction of regulatory T cells by LSECs [60], and the clonal deletion of activated T cells in the liver [61].

Tumeh and colleagues showed there was a lower density of CD8+ T cells at the invasive margins in liver versus non-liver metastases in patients with advanced melanoma in an effort to understand the liver-specific mechanisms of resistance to checkpoint inhibitor immunotherapy. They also showed that a lower density of CD8+ T cells was associated with poorer response [4]. More recently, our group compared the immune infiltrate within the tumour microenvironment of melanoma liver metastases with other metastatic sites, including lung, brain, subcutis, and lymph nodes. We described a lower density of T cells, in particular of PD1+ and CD103+ T cells, but higher Tim-3+ T cells in the tumour microenvironment of liver metastases, compared with the other sites of metastases [62]. A recent study used an in vivo colon adenocarcinoma model to demonstrate that the presence of liver metastases had a systemic immunosuppressive effect [57]. In this study, Lee and colleagues used immunocompetent C57BL/6 mice and showed that a subcutaneous tumour (MC38 cells injected subcutaneously) had a significantly higher growth in the presence of liver tumours (subcapsular injection of MC38 cells into the liver), but not in the presence of lung tumours (MC38 cells intravenously delivered into the lung). Moreover, liver tumours were less responsive to anti-PD-1 compared to subcutaneous tumours, and these subcutaneous tumours appeared less responsive to anti-PD-1 in the presence of liver tumours (while there was no difference in the presence of lung tumours), confirming that liver tumours constitute a site of resistance to immunotherapy, which negatively affects the response at distant sites of disease. In addition, the authors have shown that the presence of regulatory T cells (Tregs) was responsible for liver-specific resistance to anti-PD-1, and that by depleting these immunosuppressor cells with anti-CTLA-4 monoclonal antibodies, resistance was completely reversed. Even though the presence of Tregs is a possible liver-specific mechanism of resistance, this is not the only one in humans. Firstly, Treg depletion by anti-CTLA-4 has not been clearly shown in humans [63]. Furthermore, in patients with advanced melanoma, even though there is a subset of patients who are free of progression at 5 years (36%) when treated with the combination PD1+CTLA4 [64], 64% of patients still progress with this therapy.

Hypoxia, defined as low oxygen tensions, is a hallmark of the tumour microenvironment across cancers, which leads to local immunosuppression [65]. In hypoxic conditions, HIF1α stabilizes and binds to HIF1β, which induces the transcription of several angiogenic factors responsible for abnormal vascularization, including vascular endothelial growth factor, angiopoietin-2 (ANGPT-2), and IL-8, amongst others [66,67]. These factors have been postulated to inhibit the normal differentiation of key anti-tumour immune cells (e.g., dendritic cells) [68]. This has been clearly shown in the context of HCC, where HIF1 induces the overexpression of ectonucleoside triphosphate diphosphohydrolase 2 (ENTPD2/CD39L1) in cancer cells, which impairs the myeloid-derived suppressor cell differentiation, leading to their accumulation in the tumour microenvironment [12]. One way of overcoming hypoxia is by normalizing the vessels that feed the tumour with anti-VEGF agents, which has been successfully used in HCC and other cancers, such as CRC [19] and RCC [17]. Little work has been conducted regarding the role of hypoxia in liver metastases across cancers. Our group has recently shown that T cells are excluded from hypoxic areas within melanoma liver, lung, and subcutaneous metastases (glucose transporter 1 [Glut1] positive), which was not seen in other sites of metastases, such as brain and lymph node metastases [69]. Nevertheless, the impact of adding VEGFi to standard treatment for liver metastases across cancer types is yet to be studied. Why VEGFi may be more efficacious in patients with liver metastases is unclear. Since the liver receives a dual blood supply, in contrast to other organs, it appears that mechanisms beyond hypoxia may play a role.

Hepatocellular carcinoma is known to have a hypoxic and immunosuppressive tumour microenvironment [70], and to be resistant to chemotherapy, but responsive to VEGFi. In a phase III trial (SHARP trial) comparing sorafenib with placebo in patients with advanced HCC, there was a significant difference in time to radiologic progression (5.5 months vs. 2.8 months) and in OS (10.7 months vs. 7.9 months) favouring the sorafenib arm [8]. In another phase III trial, comparing lenvatinib with sorafenib in patients with unresectable HCC, lenvatinib showed non-inferiority in overall survival compared to sorafenib (13.6 months vs. 12.3 months) [9]. More recently, the IMBrave150 trial compared the combination of bevacizumab and atezolizumab (anti-programmed cell death ligand 1 (anti-PD-L1)) with sorafenib in unresectable HCC, and showed that the combination was associated with better clinical outcomes, including PFS (6.8 months vs. 4.3 months) and OS (not reached vs. 13.2 months) [10].

Enhancing our understanding of the potential mechanisms underlying the response to VEGFi and how these mechanisms can influence other processes, like tumour hypoxia and cell signalling, may open up opportunities for novel therapeutic agents. These agents may be aimed at targeting tumour hypoxia (e.g., targeting HIF1α pathways) or cell signalling pathways. Sanguinarine is one of such novel agents, which inhibits VEGF, induces AKT phosphorylation, and reduces angiogenesis [71,72].

From the 25 selected studies included in this meta-analysis, not all of them had PFS and OS data, which constitutes a limitation. Further to this, only a subset of these studies provided data on patients with and without liver metastases. The authors note a high degree of heterogeneity across several of the subgroup analyses, which is a limitation of this study; however, the overall heterogeneity across all studies was moderate. Such heterogeneity is not unexpected given the differences in studies, including differences in cancer type, the backbone of systemic therapy, the line of treatment, and the VEGFi type. Nevertheless, we performed subgroup analysis, and observed that the addition of VEGFi to the backbone of systemic therapy in patients with liver metastases consistently improved PFS across all subsets of patients, and there were trends, with the majority being statistically significant, towards better OS across these subgroups of patients.

## 5. Conclusions

VEGFi added to standard systemic therapy or BSC showed promising results in patients with liver metastases. For patients with liver metastases resistant to standard systemic therapy, such as checkpoint inhibitor immunotherapy, these findings suggest VEGFi may be an appropriate target as a further line of systemic therapy. This study specifically emphasizes VEGFi as a potential treatment choice, particularly for patients with liver metastases, regardless of primary tumour type, who might otherwise face an increased risk of developing resistance to standard-of-care therapy options. Translational studies are ongoing to address this and understand the biological basis of this response, and also to better identify patients with liver metastases who are resistant to standard treatment but responsive to VEGFi.

## Figures and Tables

**Table 1:** Randomized clinical trial characteristics.

Trial	NCT ID ^1^ Number Trial Phase	Cancer Type	Backbone Treatment Type	VEGF ^2^ Inhibitor (Dose)	1st Line Treatment	Liver Metastases Only	Number of Patients with Liver Metastases	PFS ^3^ HR ^4^ (95%CI)	OS ^5^ HR ^4^ (95% CI)
Escudier et al. JCO 2010 (AVOREN) [43]	NCT02056587 III	Renal Cell Carcinoma	Immunotherapy	Bevacizumab (10 mg/kg IV q2weekly)	Yes	No	138		1.61 (1.09–2.37)
Rini et al. JCO 2010 (CALGB 90206) [44]	NCT00072046 III	Renal Cell Carcinoma	Immunotherapy	Bevacizumab (10 mg/kg IV q2weekly)	Yes	No	147		0.727 (0.507–1.043)
Van Cutsem et al. JCO 2009 [47]	III	Pancreatic Cancer	Chemotherapy + Targeted therapy	Bevacizumab (5 mg/kg IV q2weekly)	Yes	No	462		0.83 (0.68–1.02)
Mir et al. Lancet Oncology 2016 (PAZOGIST) [48]	NCT01323400 II	GIST	Best supportive care	Pazopanib (800 mg PO OD)	No	No	34	0.29 (0.13–0.67)	
Fuchs et al. Lancet Oncology 2019 (RAINFALL) [49]	NCT02314117 III	Gastric orJunctional Adenocarcinoma	Chemotherapy	Ramucirumab (8 mg/kg IV D1,8 q3weekly)	Yes	No	236 ^6^	0.605 (0.433–0.847)	0.907 (0.674–1.219)
Petrylak et al. JCO 2016 [45]	NCT01282463 II	Urothelial Carcinoma	Chemotherapy	Ramucirumab (10 mg/kg IV q3weekly)	No	No	28	0.59 (0.25–1.41)	0.88 (0.39–1.96)
Petrylak et al. Lancet Oncology 2020 (RANGE) [46]	NCT02426125 III	Urothelial Carcinoma	Chemotherapy	Ramucirumab (10 mg/kg IV q3weekly)	No	No	147		0.885 (0.614–1.276)
Nakagawa et al. Lancet Oncology 2019 (RELAY) [37]	NCT02411448 III	Non-small Cell Lung Cancer	Targeted therapy	Ramucirumab (10 mg/kg IV q2weekly)	Yes	No	45	0.48 (0.23–1.02)	
Tabernero et al. Clinical Cancer Research 2013 (RESPECT) [27]	NCT00865709 II	Colorectal Cancer	Chemotherapy	Sorafenib (400 mg PO BD)	Yes	No	160	0.86 (0.60–1.24)	1.06 (0.72–1.56)
Escudier et al. NEJM 2007 (TARGET) [18]	NCT00073307 III	Renal Cell Carcinoma	Best supportive care	Sorafenib (400 mg PO BD)	No	No	233	0.44 ^7^ (0.29–0.68)	
Sandler et al. NEJM 2006. (NCT00021060) [38]	NCT00021060 II/III	Non-small Cell Lung Cancer	Chemotherapy	Bevacizumab (15 mg/kg IV q3weekly)	Yes	No	163		0.68 (0.49–0.96)
Scagliotti et al. JCO 2012 [39]	NCT00457392 III	Non-small Cell Lung Cancer	Targeted therapy	Sunitinib (37.5 mg PO OD)	No	No	182	0.957 (0.689–1.329)	0.980 (0.711–1.351)
Cunningham et al. Lancet Oncology 2013. (AVEX) [28]	NCT00484939 III	Colorectal Cancer	Chemotherapy	Bevacizumab (7.5 mg/kg IV q3weekly)	Yes	Yes	106	0.54 (0.35–0.83)	
Tabernero et al. EJC 2014 (VELOUR) [29]	NCT00561470 III	Colorectal Cancer	Chemotherapy	Aflibercept (4 mg/kg IV q2weekly)	No	Yes	299	0.547 (0.413–0.725)	0.649 (0.492–0.855)
Tang et al. JCO 2020 (BECOME) [30]	NCT01972490IV	Colorectal Cancer	Chemotherapy	Bevacizumab (5 mg/kg IV q2weekly)	Yes	Yes	241	0.49 (0.38–0.65)	0.71 (0.52–0.97)
Tebbutt et al. JCO 2010 (MAX) [31]	ACTRN12605000025639	Colorectal Cancer	Chemotherapy	Bevacizumab (7.5 mg/kg IV q3weekly)	Yes	Yes	61	0.25 ^8^	
Li et al. Future Oncology 2018 [32]	NCT01661270 III	Colorectal Cancer	Chemotherapy	Aflibercept4 mg/kg IV q2weekly)	No	Yes	71	0.54 (0.3–0.971)	
Tabernero et al. Lancet Oncology 2015 (RAISE) [33]	NCT01183780 III	Colorectal Cancer	Chemotherapy	Ramucirumab (8 mg/kg IV q2weekly)	No	Yes	187	0.801 (0.590–1.089)	0.963 (0.679–1.367)
Chi et al. The Oncologist 2021 (ALTER0703) [34]	NCT02332499 II/III	Colorectal Cancer	Chemotherapy	Anlotinib (12 mg PO D1-14 q3weekly)	No	No	312	0.27 (0.20–0.36)	0.92 (0.71–1.2)
Doebele et al. Cancer 2015 [40]	NCT01160744 II	Non-small Cell Lung Cancer	Chemotherapy	Ramucirumab (10 mg/kg IV q3weekly)	Yes	No	24	0.45 ^9^ (0.25–1.05)	
Li et al. Jama 2018 (FRESCO) [35]	NCT02314819 III	Colorectal Cancer	Best supportive care	Fruquintinib (5 mg PO OD D1-21 q3weekly)	No	No	287	0.22 (0.17–0.30)	0.59 (0.45–0.77)
Van Cutsem et al. Annals of Oncology 2018 (LUME-Colon 1) [36]	NCT02149108 III	Colorectal Cancer	Best supportive care	Nintedanib (200 mg PO BD)	No	No	543	0.53 (0.44–0.64)	0.95 (0.79–1.14)
Zhao et al. Journal of Thoracic Oncology 2021 (CTONG1706) [41]	NCT02824458 III	Non-small Cell Lung Cancer	Targeted therapy	Apatinib (500 mg PO OD)	Yes	No	40	0.42 (0.15–1.17)	
Kim et al. JCO 2012 (BEAM) [50]	NCT00434252. II	Melanoma	Chemotherapy	Bevacizumab (15 mg/kg IV q3weekly)	Yes	No	96	0.73 (0.46–1.16)	0.60 (0.36–1.00)
Shen et al. Journal of Cancer Research and Clinical Oncology 2013 (ALTER 0303) [42]	NCT02388919 III	Non-small Cell Lung Cancer	Best supportive care	Anlotinib (12 mg PO OD)	No	No	78	0.23 (0.12–0.42)	0.61 (0.36–1.02)

^1^ NCT ID number, National Clinical Trials (NCT) identification number; ^2^ VEGF, vascular endothelial growth factor; ^3^ PFS, progression-free survival; ^4^ HR, hazard ratio; ^5^ OS, overall survival; ^6^ Fuchs et al., Lancet Oncology 2019 (RAINFALL)—number of patients with liver metastasis reported in OS analysis n = 236; number of patients with liver metastasis reported in PFS n = 189. ^7^ Escudier et al. NEJM 2007 (TARGET)—HR estimated from Figure 3 of the manuscript. ^8^ Tebbutt et al. JCO 2010 (MAX)—95% CI for HR for PFS was not provided in the manuscript. ^9^ Doebele et al. Cancer 2015—HR estimated from Figure 4 of the manuscript.

## Data Availability

The data presented in this study are available in this article.

## Supplementary Materials

The following supporting information can be downloaded at: https://www.mdpi.com/article/10.3390/cancers15205012/s1, Table S1: VEGF inhibitors. Table S2: Search Strategy. Table S3: RCT quality assessment. Figure S1: The addition of VEGFi to a backbone of systemic therapy or BSC was associated with superior PFS in patients with liver metastases from “colorectal” and “non-colorectal” cancers. Forest plot and pooled HRs for PFS comparing the backbone systemic therapy or BSC with versus without VEGFi in patients with liver metastases from “colorectal cancer” (HR = 0.48; 95% CI, 0.34–0.68; high heterogeneity: I^2^ = 89%, *p* < 0.001) (**A**) and with liver metastases from “non-colorectal cancer” (GIST, gastric or junctional adenocarcinoma, urothelial carcinoma, non-small cell lung cancer, renal cell carcinoma, melanoma) (HR = 0.51; 95% CI, 0.39–0.68; high heterogeneity: I^2^ = 62%, *p* = 0.005) (**B**). Figure S2: The addition of VEGFi to “chemotherapy” and “non-chemotherapy” was associated with superior PFS in patients with liver metastases across cancers. Forest plot and pooled HRs for PFS comparing “chemotherapy” with versus without VEGFi in patients with liver metastases across cancers (HR = 0.63; 95% CI, 0.53–0.75; low heterogeneity: I^2^ = 25, *p* = 0.22) (**A**) and “non-chemotherapy” (non-VEGFi targeted therapy or BSC) with versus without VEGFi in patients with liver metastases across cancers (HR = 0.39; 95% CI, 0.27–0.55; high heterogeneity: I^2^ = 87%, *p* < 0.001) (**B**). Figure S3: The addition of VEGFi (“bevacizumab” and “non-bevacizumab”) to a backbone of systemic therapy or BSC was associated with superior PFS in patients with liver metastases across cancers. Forest plot and pooled HRs for PFS comparing a backbone of systemic therapy with versus without VEGFi (“bevacizumab”) in patients with liver metastases across cancers (HR = 0.54; 95% CI, 0.44–0.67; low heterogeneity: I^2^ = 6%, *p* = 0.34) (**A**) and with versus without VEGFi (“non-bevacizumab” [pazopanib, ramucirumab, sorafenib, sunitinib, aflibercept, anlotinib, fruquintinib, nintedanib, apatinib) in patients with liver metastases across cancers (HR = 0.48; 95% CI, 0.37–0.62; high heterogeneity: I^2^ = 85%, *p* < 0.001) (**B**). Figure S4: The addition of VEGFi to a backbone of systemic therapy or BSC as “1st line” and “subsequent line” of treatment was associated with superior PFS in patients with liver metastases across cancers. Forest plot and pooled HRs for PFS comparing a backbone of systemic therapy with versus without VEGFi as “1st line treatment” in patients with liver metastases across cancers (HR = 0.62; 95% CI, 0.52–0.74; low heterogeneity: I^2^ = 30%, *p* = 0.18) (**A**) and as “subsequent line treatment” in patients with liver metastases across cancers (HR = 0.40; 95% CI, 0.29–0.57; high heterogeneity: I^2^ = 88%, *p* < 0.001) (**B**). Figure S5: The addition of VEGFi to a backbone of systemic therapy or BSC was associated with superior OS in patients with liver metastases from “colorectal” and “non-colorectal” cancers. Forest plot and pooled HRs for OS comparing the backbone systemic therapy or BSC with versus without VEGFi in patients with liver metastases from “colorectal cancer” (HR = 0.81; 95% CI, 0.69–0.96; moderate heterogeneity: I^2^ = 60%, *p* = 0.02) (**A**) and with liver metastases from “non-colorectal cancer” (GIST, gastric or junctional adenocarcinoma, urothelial carcinoma, non-small cell lung cancer, renal cell carcinoma, melanoma) (HR = 0.85; 95%CI, 0.72–1.00; moderate heterogeneity: I^2^ = 48%, *p* = 0.05) (**B**). Figure S6: The addition of VEGFi to “chemotherapy” and “non-chemotherapy” was associated with superior OS in patients with liver metastases across cancers. Forest plot and pooled HRs for OS comparing “chemotherapy” with versus without VEGFi in patients with liver metastases across cancers (HR = 0.79; 95% CI, 0.70–0.91; low heterogeneity: I^2^ = 11%, *p* = 0.34) (**A**) and “non-chemotherapy” (targeted therapy or BSC) with versus without VEGFi in patients with liver metastases across cancers (HR = 0.86; 95% CI, 0.71–1.05; high heterogeneity: I^2^ = 68%, *p* = 0.002) (**B**). Figure S7: The addition of VEGFi (“bevacizumab” and “non-bevacizumab”) to a backbone of systemic therapy or BSC was associated with superior OS in patients with liver metastases across cancers. Forest plot and pooled HRs for OS comparing a backbone of systemic therapy with versus without VEGFi (“bevacizumab”) in patients with liver metastases across cancers (HR = 0.81; 95% CI, 0.63–1.05; high heterogeneity: I^2^ = 68%, *p* = 0.009) (**A**) and with versus without VEGFi (“non-bevacizumab” [pazopanib, ramucirumab, sorafenib, sunitinib, aflibercept, anlotinib, fruquintinib, nintedanib, apatinib) in patients with liver metastases across cancers (HR = 0.84; 95% CI, 0.74–0.96; moderate heterogeneity, I^2^ = 41%, *p* = 0.08) (**B**). Figure S8: The addition of VEGFi to a backbone of systemic therapy or BSC as 1st or subsequent line of treatment was associated with superior OS in patients with liver metastases across cancers. Forest plot and pooled HRs for OS comparing a backbone of systemic therapy with versus without VEGFi as “1st line treatment” in patients with liver metastases across cancers (HR = 0.86; 95% CI, 0.73–1.02; moderate heterogeneity: I^2^ = 55%, *p* = 0.02) (**A**) and as “subsequent line treatment” in patients with liver metastases across cancers (HR = 0.80; 95% CI, 0.68–0.94; moderate heterogeneity: I^2^ = 51%, *p* = 0.05) (**B**).
