# Supplementary material for: VEGF Inhibitors Improve Survival Outcomes in Patients with Liver Metastases across Cancer Types—A Meta-Analysis
Source: Cancers (Basel). 2023 Oct 16;15(20):5012. doi: 10.3390/cancers15205012 (PMC10605052; doi:10.3390/cancers15205012)
Supplement: Supplementary file 1 [file cancers-15-05012-s001.zip › cancers-2615458-supplementary.pdf]

Supplementary Material

Supplementary Table S1. VEGF inhibitors.

Name of VEGFi	Agent type	Target
**Sunitinib**	TKI	VEGFR-1,-2, and -3, PDGFR, c-Kit, Flt-3, CSFR1, glial cell-derived neurotrophic factor receptor
**Pazopanib**	TKI	VEGFR-1,-2,-3, PDGFR-a and -ß, c-Kit
**Sorafenib**	TKI	VEGFR-2, VEGFR-3, PDGFR-ß, c-KIT, FLT-3, CRAF, BRAF and mutant BRAF,
**Lenvatinib**	TKI	VEGFR-1,-2, and -3, FGFR-1, FGFR-2,-3, and -4, PDGFRa, RET, and c-KIT
**Vandetanib**	TKI	VEGFR-2, EGFR
**Regorafenib**	TKI	VEGFR-1,-2, and -3, TIE-2, c-KIT, RET, RAF1, BRAF, and BRAFV600E, FDGFR, FGFR
**Cabozantinib**	TKI	VEGFR-1,-2 and -3, c-KIT, TRKB, Flt-3, AXL, RET, MET, and TIE-2
**Axitinib**	TKI	VEGFR-1,-2, and -3
**Cediranib**	TKI	VEGFR-1,-2, and -3
**Ponatinib**	TKI	VEGFR, BCR-ABL
**Aflibercept**	Soluble decoy receptor	VEGF A-D, PIGF
**Vatalanib**	TKI	VEGFR, PDGFR, c-KIT
**Tivozanib**	TKI	VEGFR-1,-2, and -3, PDGFR-ß
**Motesanib**	TKI	VEGFR, PDGFR
**Linifanib**	TKI	VEGFR
**Anlotinib**	TKI	VEGFR, FGFR, and PDGFR
**Fruquintinib**	TKI	VEGFR-1,-2, and -3
**Nintedanib**	TKI	VEGFR, FGFR, and PDGFR
**Apatinib**	TKI	VEGFR-2, c-KIT, c-SRC
**Bevacizumab**	Monoclonal antibody	VEGF
**Ramucirumab**	Monoclonal antibody	VEGFR-2

**Vanucizumab**	Monoclonal antibody	VEGF, angiopoietin-2

Supplementary Table S2. Search Strategy.

Database	Keywords	Number of studies
**Ovid Medline**		
**1**	Neoplasms/	497186
**2**	exp Carcinoma/	723044
**3**	exp Neoplasm Metastasis/	221165
**4**	(Neoplas* or cancer* or tumour* or tumor* or malignan* or carcinoma* or adenocarcinoma* or stage four cancer* or metast*).mp.	4876470
**5**	Sunitinib/	4133
**6**	Angiogenesis Inhibitors/	28867
**7**	Sorafenib/	6224
**8**	Bevacizumab/	14127
**9**	Axitinib/	716
**10**	Response*.mp.	3499677
**11**	disease-free survival/ or progression-free survival/ or response evaluation criteria in solid tumors/	89668
**12**	(disease?free survival* or progression?free survival* or response evaluation criteria in solid tumo?r* or RECIST or Overall survival*).mp.	236936
**13**	Randomized Controlled Trials as Topic/	162013
**14**	randomi?ed controlled trial.pt.	592772
**15**	(Stage 4 cancer* or advance* cancer* or Metast*).mp.	703445
**16**	control* trial.kw.	302
**17**	(sunitinib or pazopanib or vendetanib or lenvatanib or regorafenib or cabozantinib or cediranib or ponatanib or aflibercept or vatalanib or tivozanib or ramucirumab or motesanib).tw.	14697

**18**	1 or 2 or 3 or 4 or 15	4877863
**19**	5 or 6 or 7 or 8 or 9 or 17	52148
**20**	5 or 7 or 8 or 9 or 17	33872
**21**	10 or 11 or 12	3712762
**22**	13 or 14 or 16	749128
**23**	18 and 19 and 21 and 22	2062
**24**	18 and 20 and 21 and 22	1900
**25**	limit 24 to (humans and clinical trial, all and "therapy (maximizes sensitivity)" and medline)	1415
**COCHRANE CENTRAL**		
**1**	exp Neoplasms/	89556
**2**	(cancer or onco* or tumour or tumor).mp.	229437
**3**	exp Neoplasm Metastasis/	5568
**4**	(stage four cancer* or metast* or advanc* canc* or stage 4 cancer*).mp.	59226
**5**	exp Sunitinib/	357
**6**	exp Sorafenib/	541
**7**	exp Bevacizumab/	2223
**8**	exp Axitinib/	110
**9**	(disease?free survival* or progression?free survival* or response evaluation criteria in solid tumo?r* or RECIST or Overall survival* or DFS or PFS or OS).mp.	67468
**10**	1 or 2 or 3 or 4	255360
**11**	(sunitinib or pazopanib or vendetanib or lenvatanib or regorafenib or cabozantinib or cediranib or ponatanib or aflibercept or vatalanib or tivozanib or ramucirumab or motesanib or anlotinib or fruquitinib).tw.	4571

**12**	9 and 10 and 11	2511
**13**	limit 12 to (clinical trial or clinical trial, phase i or clinical trial, phase ii or clinical trial, phase iii or clinical trial, phase iv or controlled clinical trial or randomized controlled trial)	453
**EMBASE**		
**1**	exp malignant neoplasm/	4073600
**2**	exp metastasis/	809335
**3**	(stage four cancer* or advanc* canc* or stage 4 cancer*).mp.	173583
**4**	exp sunitinib/	27348
**5**	exp sorafenib/	37394
**6**	exp bevacizumab/	72775
**7**	exp axitinib/	7085
**8**	exp pazopanib/	10590
**9**	exp regorafenib/	6617
**10**	exp cabozantinib/	6655
**11**	exp aflibercept/	8859
**12**	exp ramucirumab/	4532
**13**	exp motesanib/	838
**14**	exp linifanib/	602
**15**	(vendetanib or lenvatanib or cediranib or ponatanib or vatalanib or tivozanib).tw.	1247
**16**	exp disease free survival/	111754
**17**	exp overall survival/	465372
**18**	exp progression free survival/	166293
**19**	(disease?free survival* or progression?free survival* or response evaluation criteria in solid tumo?r* or RECIST or Overall survival* or DFS or PFS or OS).mp.	675343
**20**	response evaluation criteria in solid tumors/	18365

**21**	1 or 2 or 3	4166621
**22**	4 or 5 or 6 or 7 or 8 or 9 or 10 or 11 or 12 or 13 or 14 or 15	130727
**23**	16 or 17 or 18 or 19 or 20	733941
**23**	21 and 22 and 23	43832
**24**	limit 24 to (human and (clinical trial or randomized controlled trial or controlled clinical trial or multicenter study or phase 1 clinical trial or phase 2 clinical trial or phase 3 clinical trial or phase 4 clinical trial) and "therapy (maximizes sensitivity)" and article and journal)	4856

Supplementary Table S3. RCT quality assessment.

Trial	Randomization	Blinding	Withdrawals and dropouts	Total Jadad Scale
**Escudier *et al*. JCO 2010 (AVOREN)[43]**	2	2	1	**5**
**Rini *et al*. JCO 2010 (CALGB 90206)[44]**	2	0	1	**3**
**Van Cutsem *et al.* JCO 2009[47]**	2	2	1	**5**
**Mir *et al.* Lancet Oncology 2016 (PAZOGIST)[48]**	2	0	1	**3**
**Fuchs *et al.* Lancet Oncology 2019 (RAINFALL)[49]**	2	2	1	**5**
**Petrylak *et al.* JCO 2016[45]**	2	0	1	**3**
**Petrylak *et al.* Lancet Oncology 2020 (RANGE)[46]**	2	2	1	**5**
**Nakagawa *et al.* Lancet Oncology 2019 (RELAY)[37]**	2	2	1	**5**
**Tabernero *et al.* Clinical Cancer Research 2013 (RESPECT)[27]**	2	2	1	**5**
**Escudier *et al.* NEJM 2007 (TARGET)[18]**	2	2	1	**5**
**Sandler *et al.* NEJM 2006. (NCT00021060)[38]**	2	0	1	**3**
**Scagliotti *et al.* JCO 2012**	2	2	1	**5**
**Cunningham *et al.* Lancet Oncology 2013 (AVEX)[28]**	2	0	1	**3**
**Tabernero *et al.* EJC 2014 (VELOUR)[29]**	2	2	1	**5**
**Tang *et al.* JCO 2020 (BECOME)[30]**	2	0	1	**3**
**Tebbutt *et al.* JCO 2010 (MAX)[31]**	2	0	1	**3**
**Li *et al.* Future Oncology 2018[32]**	2	2	1	**5**
**Tabernero *et al.* Lancet Oncology 2015 (RAISE)[33]**	2	2	1	**5**
**Chi *et al.* The Oncologist 2021 (ALTER0703)[34]**	2	2	1	**5**
**Doebele *et al.* Cancer 2015[40]**	2	0	1	**3**
**Li *et al.* Jama 2018 (FRESCO)[35]**	2	2	1	**5**
**Van Cutsem *et al.* Annals of Oncology 2018 (LUME-Colon 1)[36]**	2	2	0	**4**
**Zhao *et al.* Journal of Thoracic Oncology 2021 (CTONG1706)[41]**	2	2	1	**5**
**Kim *et al.* JCO 2012 (BEAM)[50]**	2	2	1	**5**
**Shen *et al.* Journal of Cancer Research and Clinical Oncology 2023 (ALTER 0303)[42]**	2	2	0	**4**

A

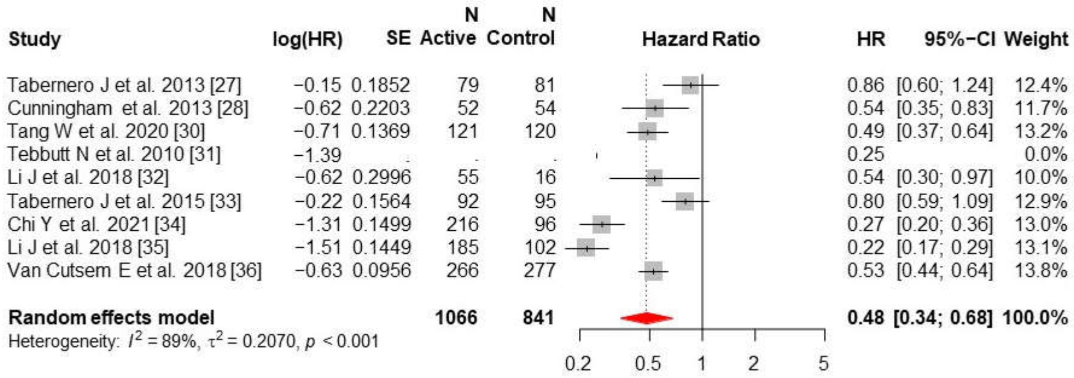

B

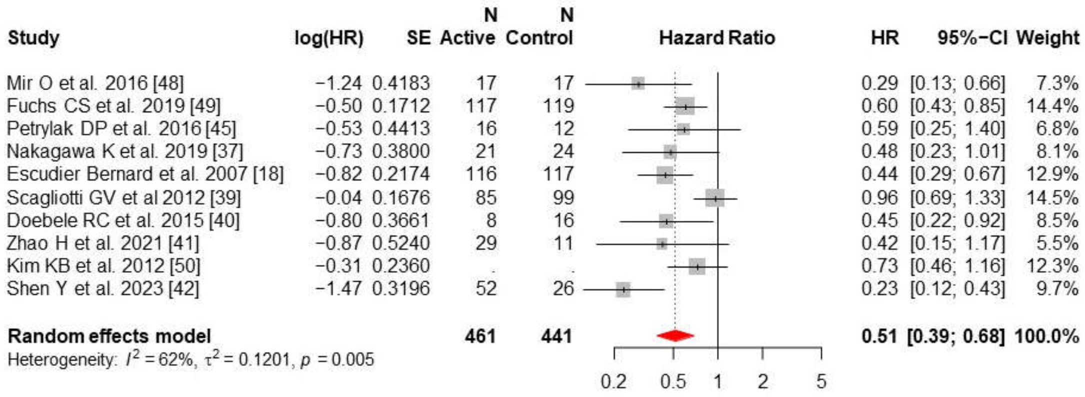

Supplementary Figure S1. The addition of VEGFi to a backbone of systemic therapy or BSC was associated with superior PFS in patients with liver metastases from “*colorectal*” and “*non-colorectal*” cancers. Forest plot and pooled HRs for PFS comparing the backbone systemic therapy or BSC with versus without VEGFi in patients with liver metastases from “*colorectal cancer*” (HR = 0.48; 95% CI, 0.34 - 0.68; high heterogeneity: $I^2 = 89\%$, p < 0.001) **(A)** and with liver metastases from “*non-colorectal cancer*” (GIST, gastric or junctional adenocarcinoma, urothelial carcinoma, non-small cell lung cancer, renal cell carcinoma, melanoma) (HR = 0.51; 95% CI, 0.39 - 0.68; high heterogeneity: $I^2 = 62\%$, p = 0.005) **(B)**.

A

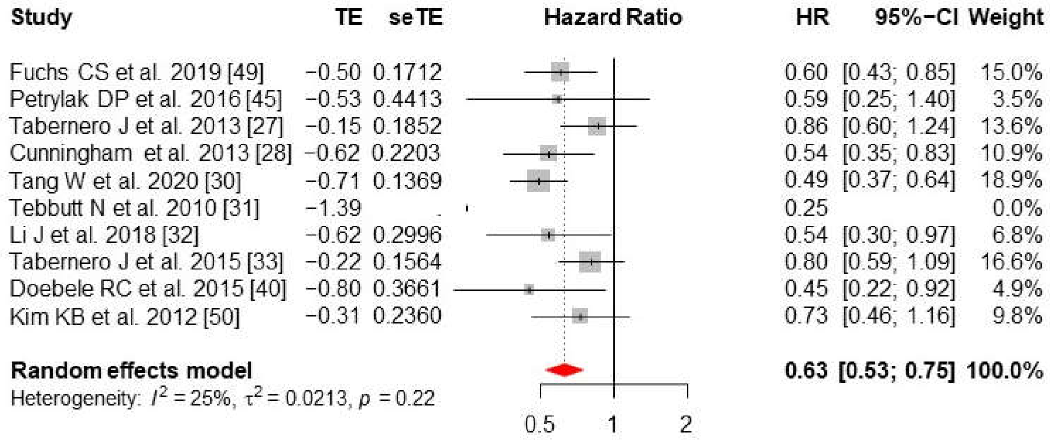

B

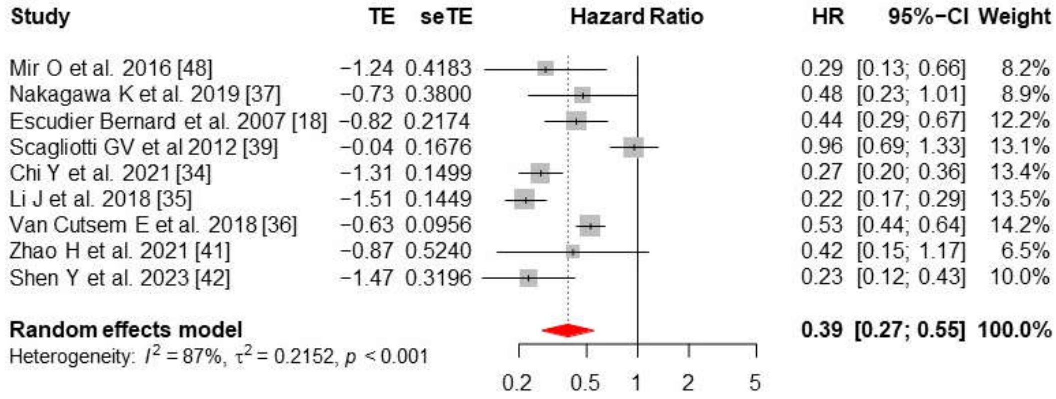

Supplementary Figure S2. The addition of VEGFi to "*chemotherapy*" and "*non-chemotherapy*" was associated with superior PFS in patients with liver metastases across cancers. Forest plot and pooled HRs for PFS comparing "*chemotherapy*" with versus without VEGFi in patients with liver metastases across cancers (HR = 0.63; 95% CI, 0.53 – 0.75; low heterogeneity: I^2 = 25, p =

0.22) **(A)** and “*non-chemotherapy*” (non-VEGFi targeted therapy or BSC) with versus without VEGFi in patients with liver metastases across cancers (HR = 0.39; 95% CI, 0.27 - 0.55; high heterogeneity: I^2 = 87%, p < 0.001) **(B)**.

A

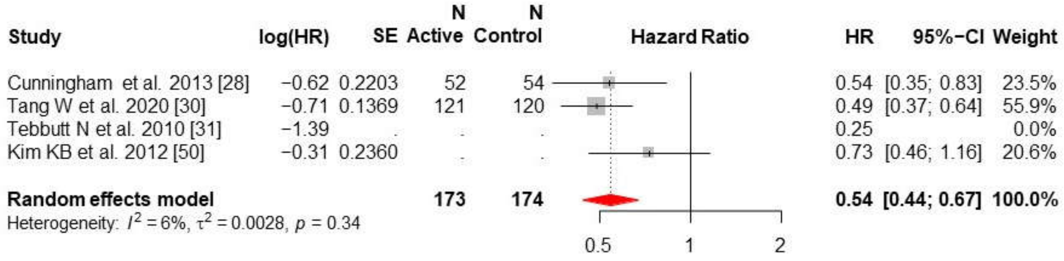

B

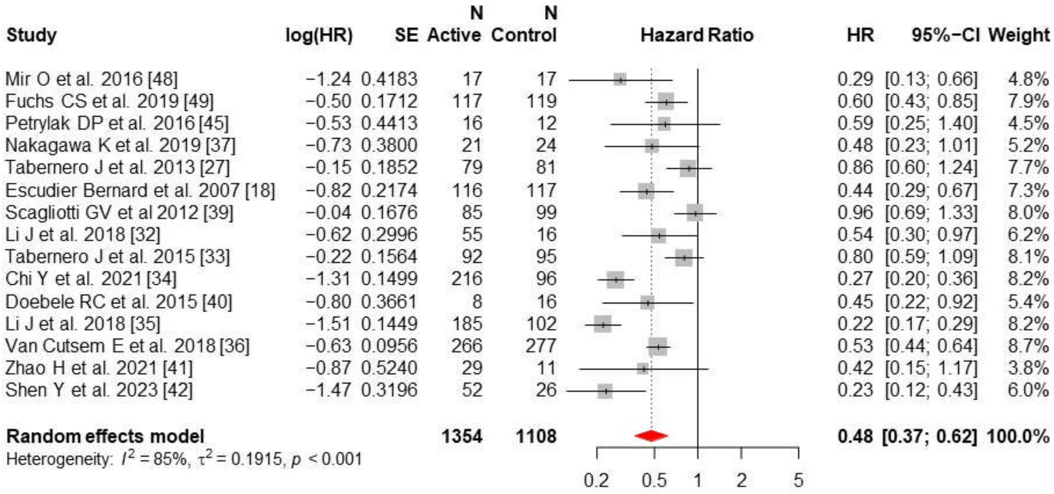

Supplementary Figure S3. The addition of VEGFi (“*bevacizumab*” and “*non-bevacizumab*”) to a backbone of systemic therapy or BSC was associated with superior PFS in patients with liver metastases across cancers. Forest plot and pooled HRs for PFS comparing a backbone of systemic therapy with versus without VEGFi (“*bevacizumab*”) in patients with liver metastases across cancers (HR = 0.54; 95% CI, 0.44 – 0.67; low heterogeneity: I^2 = 6%, p = 0.34) **(A)** and with versus without VEGFi (“*non-bevacizumab*” [pazopanib, ramucirumab,

sorafenib, sunitinib, aflibercept, anlotinib, fruquintinib, nintedanib, apatinib) in patients with liver metastases across cancers (HR = 0.48; 95% CI, 0.37 – 0.62; high heterogeneity: I^2 = 85%, p < 0.001) **(B).**

A

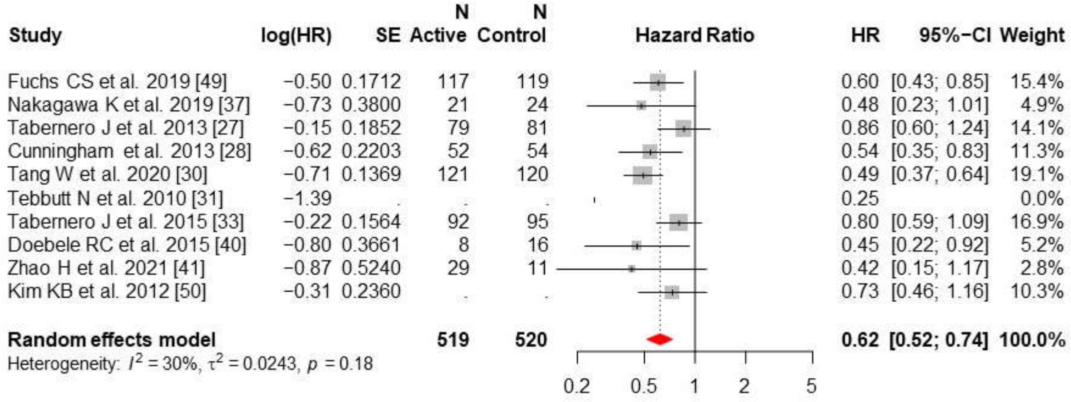

B

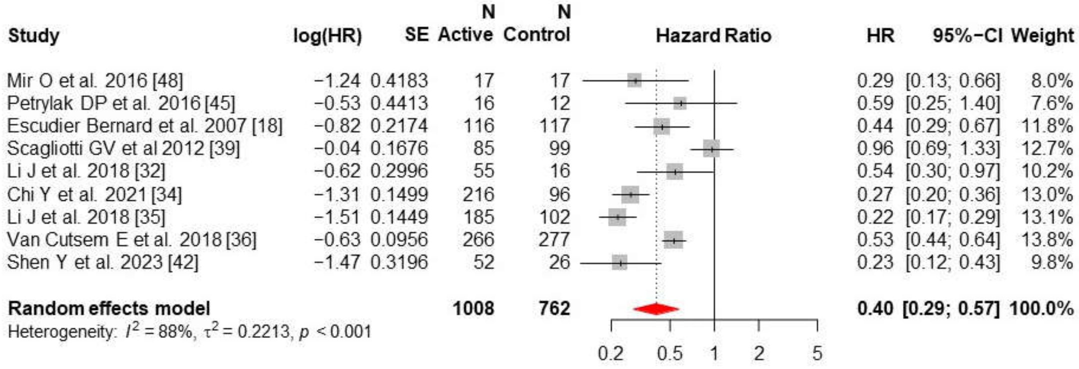

Supplementary Figure S4. The addition of VEGFi to a backbone of systemic therapy or BSC as "*1st line" and "subsequent line" of treatment* was associated with superior PFS in patients with liver metastases across cancers. Forest plot and pooled HRs for PFS comparing a backbone of systemic therapy with versus without VEGFi as "*1st line treatment*" in patients with liver metastases across

cancers (HR = 0.62; 95% CI, 0.52 – 0.74; low heterogeneity: I^2 = 30%, p = 0.18) **(A)** and as "*subsequent line treatment*" in patients with liver metastases across cancers (HR = 0.40; 95% CI, 0.29 – 0.57; high heterogeneity: I^2 = 88%, p < 0.001) **(B)**.

A

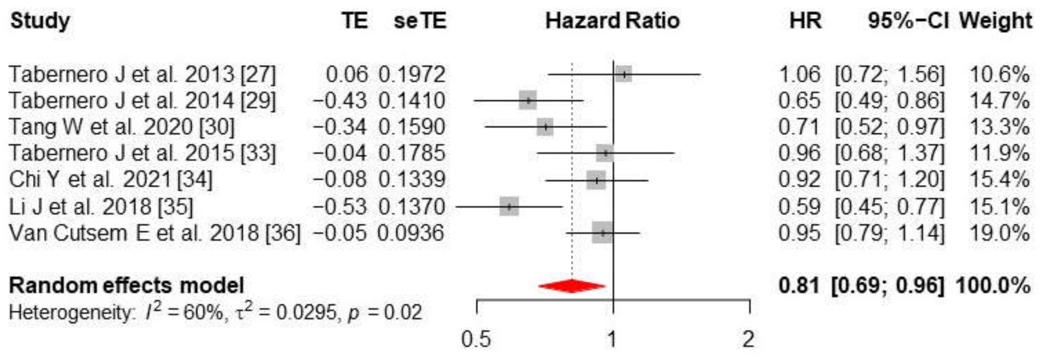

B

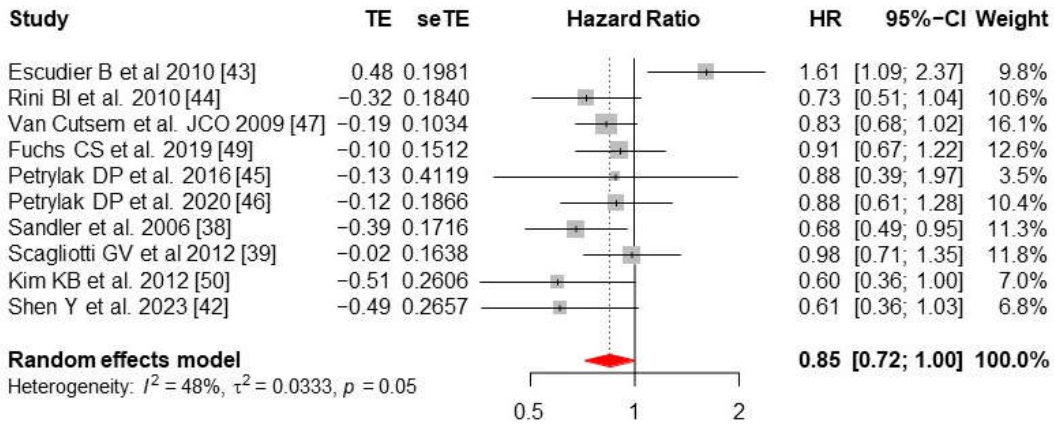

Supplementary Figure S5. The addition of VEGFi to a backbone of systemic therapy or BSC was associated with superior OS in patients with liver metastases from "*colorectal*" and "*non-colorectal*" cancers. Forest plot and pooled HRs for OS comparing the backbone systemic therapy or BSC with versus without VEGFi in patients with liver metastases from "*colorectal cancer*" (HR = 0.81; 95% CI, 0.69 - 0.96; moderate heterogeneity: I^2 = 60%, p = 0.02) **(A)** and with liver metastases from "*non-colorectal cancer*" (GIST, gastric or junctional

adenocarcinoma, urothelial carcinoma, non-small cell lung cancer, renal cell carcinoma, melanoma) (HR = 0.85; 95%CI, 0.72 – 1.00; moderate heterogeneity: I^2 = 48%, p = 0.05) **(B)**.

A

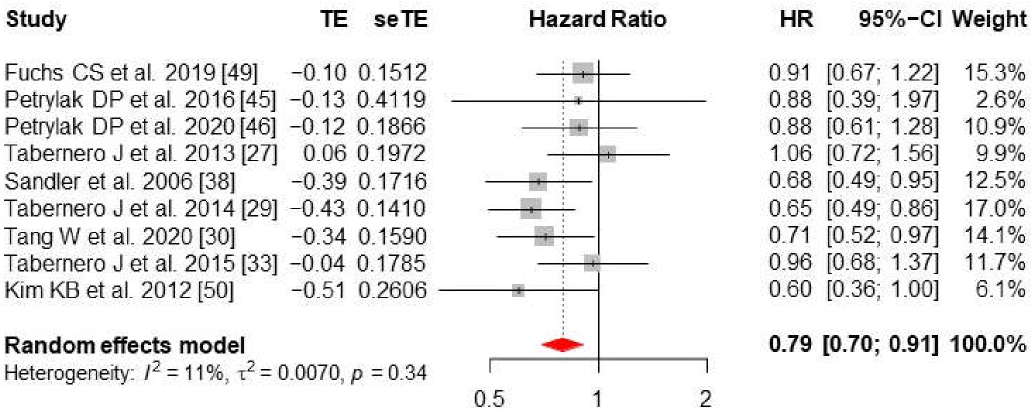

B

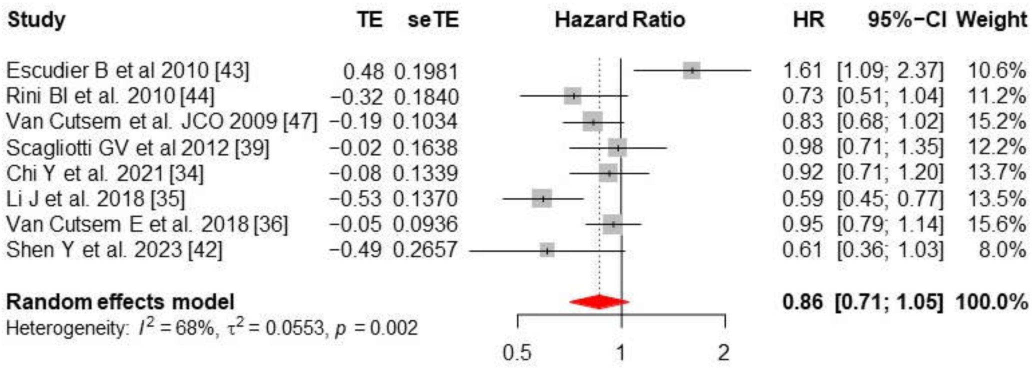

Supplementary Figure S6. The addition of VEGFi to "*chemotherapy*" and "*non-chemotherapy*" was associated with superior OS in patients with liver metastases across cancers. Forest plot and pooled HRs for OS comparing "*chemotherapy*" with versus without VEGFi in patients with liver metastases across cancers (HR = 0.79; 95% CI, 0.70 – 0.91; low heterogeneity: I^2 = 11%, p = 0.34) **(A)** and "*non-*

chemotherapy" (targeted therapy or BSC) with versus without VEGFi in patients with liver metastases across cancers (HR = 0.86; 95% CI, 0.71 - 1.05; high heterogeneity: I^2 = 68%, p = 0.002) **(B)**.

A

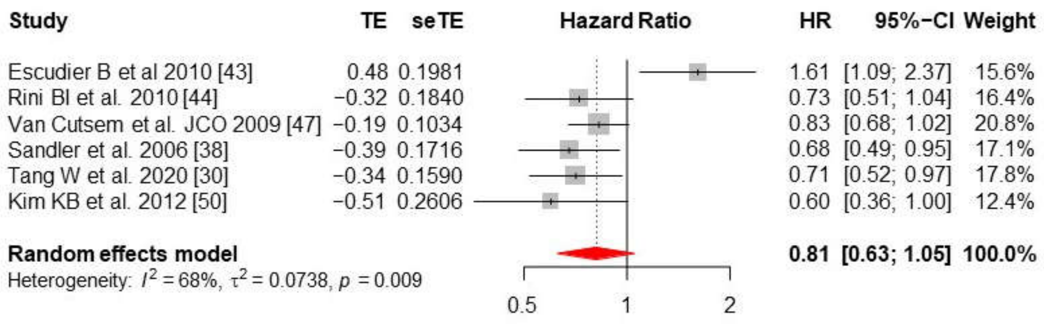

B

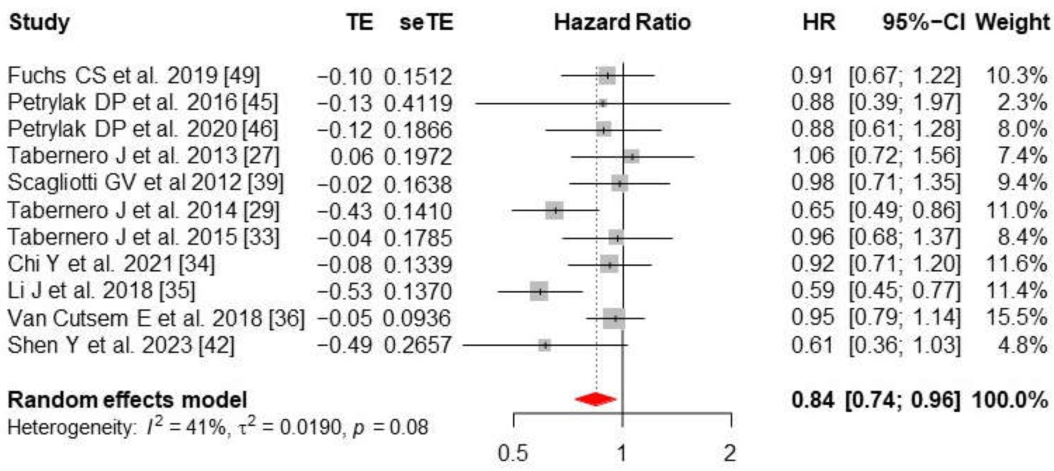

Supplementary Figure S7. The addition of VEGFi ("*bevacizumab*" and "*non-bevacizumab*") to a backbone of systemic therapy or BSC was associated with superior OS in patients with liver metastases across cancers. Forest plot and pooled HRs for OS comparing a backbone of systemic therapy with versus without VEGFi ("*bevacizumab*") in patients with liver metastases across cancers (HR = 0.81; 95% CI, 0.63 – 1.05; high heterogeneity: I^2 = 68%, p = 0.009) **(A)** and

with versus without VEGFi ("*non-bevacizumab*" [pazopanib, ramucirumab, sorafenib, sunitinib, aflibercept, anlotinib, fruquintinib, nintedanib, apatinib) in patients with liver metastases across cancers (HR = 0.84; 95% CI, 0.74 – 0.96; moderate heterogeneity, I^2 = 41%, p = 0.08) **(B).**

A

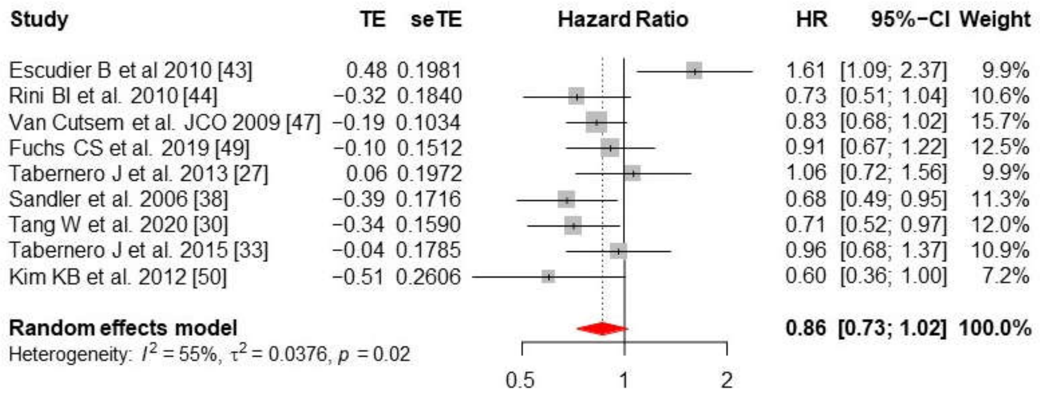

B

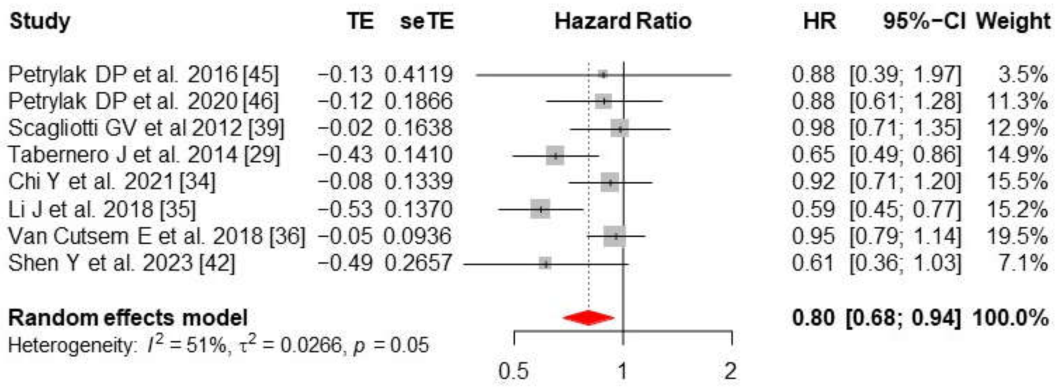

Supplementary Figure S8. The addition of VEGFi to a backbone of systemic therapy or BSC as *1st or subsequent line of treatment* was associated with superior OS in patients with liver metastases across cancers. Forest plot and pooled HRs for OS comparing a backbone of systemic therapy with versus without VEGFi as "*1st line treatment*" in patients with liver metastases across

cancers (HR = 0.86; 95% CI, 0.73 – 1.02; moderate heterogeneity: $I^2 = 55\%$, p = 0.02) **(A)** and as "*subsequent line treatment*" in patients with liver metastases across cancers (HR = 0.80; 95% CI, 0.68 – 0.94; moderate heterogeneity: $I^2 =$ 51%, p = 0.05) **(B)**.
